# Supplementary material for: How Can Out-of-Hospital Cardiac Arrest (OHCA) Data Collection in Slovenia Be Improved?
Source: Medicina (Kaunas). 2023 May 30;59(6):1050. doi: 10.3390/medicina59061050 (PMC10302052; doi:10.3390/medicina59061050)
Supplement: Supplementary file 1 [file medicina-59-01050-s001.zip › medicina-2396707-supplementary.pdf]

Petravić, L.; Burger, E.; Keše, U.; Kulovec, D.; Miklič, R.;
Poljanšek, E.; Tomšič, G.; Pintarič, T.; Lopes, M.F.; Turnšek, E.; et al. How Can Out-of-Hospital Cardiac Arrest (OHCA) Data Collection in Slovenia Be Improved? *Medicina* **2023**

Supplement 1: List of all OHCA registries found

Table S1: List of all OHCA registries found, with their respecitve name and country of origin

**Registry**	**Country**	**Source**
The Australasian Resuscitation Outcomes Consortium (Aus-ROC)	Australia and Asia	[29]
Victorian Ambulance Cardiac Arrest Registry (VACAR)	Australia Victoria	[30]
Austrian resuscitation council (ARC)	Austria	[31]
Helsinki Cardiac Arrest Registry	Finland, Helsinki	[32]
German Resuscitation Registry	Germany	[33]
Utstein Osaka Project	Japan	[34]
CArdioVAscular disease Surveillance (CAVAS) project	Korea, Seoul	[35]
Amsterdam resuscitation studies (ARREST)	Netherlands	[36]
Norwegian Cardiac Arrest Registry (NorCAR)	Norway	[37]
The Silesian Registry of Out-of-Hospital Cardiac Arrest (SIL-OHCA)	Poland	[38]
Irish Out of Hospital Cardiac Arrest Register (OHCAR)	Republic of Ireland	[39]
Swedish Register of Cardiopulmonary Resuscitation	Sweden	[40]
Taipei OHCA Registry	Taiwan Taipei	[41]

Cardiac Arrest Registry to Enhance Survival (CARES)	United states of America	[42]
Resuscitation Outcomes Consortium (ROC)	US	[43]

References:

29 Variables « Aus-ROC Available online: https://www.ausroc.org.au/epistry/variables/ (accessed on 9 April 2022).

30. Smith, K.L.; Bray, J.; Barnes, V.; Lodder, M.; Cameron, P.; Bernard, S.; Currell, A. Victorian Ambulance Cardiac Arrest Registry. *Resuscitation* **2010**, *81*, S4, doi:10.1016/j.resuscitation.2010.09.030.

31. Österreichischer Rat Für Wiederbelebung – ARC – Austrian Resuscitation Council Available online: https://www.arc.or.at/ (accessed on 9 April 2022).

32. Skrifvars, M.B.; Vayrynen, T.; Kuisma, M.; Castren, M.; Parr, M.J.; Silfverstople, J.; Svensson, L.; Jonsson, L.; Herlitz, J. Comparison of Helsinki and European Resuscitation Council "Do Not Attempt to Resuscitate" Guidelines, and a Termination of Resuscitation Clinical Prediction Rule for out-of-Hospital Cardiac Arrest Patients Found in Asystole or Pulseless Electrical Activity. *Resuscitation* **2010**, *81*, 679–684, doi:10.1016/j.resuscitation.2010.01.033.

33. Formulare | Deutsches Reanimationsregister Available online: https://www.reanimationsregister.de/downloads/formulare.html (accessed on 9 April 2022).

34. Kitamura, T.; Iwami, T.; Atsumi, T.; Endo, T.; Kanna, T.; Kuroda, Y.; Sakurai, A.; Tasaki, O.; Tahara, Y.; Tsuruta, R.; et al. The Profile of Japanese Association for Acute Medicine – out-of-Hospital Cardiac Arrest Registry in 2014–2015. *Acute Med. Surg.* **2018**, *5*, 249–258, doi:10.1002/ams2.340.

35. Kim, J.Y.; Hwang, S.O.; Shin, S.D.; Yang, H.J.; Chung, S.P.; Lee, S.W.; Song, K.J.; Hwang, S.S.; Cho, G.C.; Moon, S.W.; et al. Korean Cardiac Arrest Research Consortium (KoCARC): Rationale, Development, and Implementation. *Clin. Exp. Emerg. Med.* **2018**, *5*, 165, doi:10.15441/ceem.17.259.

36. Academic Medical Centre – ESCAPE-NET Available online: https://escape-net.eu/consortium/academic-medical-centre/ (accessed on 9 April 2022).

37. Norwegian Cardiac Arrest Registry (NorCAR) Available online: http://helsedata.no/en/forvaltere/norwegian-institute-of-public-health/norwegian-cardiac-arrest-registry/ (accessed on 9 April 2022).

38. Nadolny, K.; Bujak, K.; Kucap, M.; Trzeciak, P.; Hudzik, B.; Borowicz, A.; Gąsior, M. The Silesian Registry of Out-of-Hospital Cardiac Arrest: Study Design and Results of a Three-Month Pilot Study. *Cardiol. J.* **2020**, *27*, 566–574, doi:10.5603/CJ.a2018.0140.

39. Out of Hospital Cardiac Arrest Register (OHCAR) - NUI Galway Available online: https://www.nuigalway.ie/ohcar/# (accessed on 9 April 2022).

40. Svenska HLR-registret 2021 Available online: https://www.hlr.nu/svenska-hlr-registret/ (accessed on 9 April 2022).

41. Lin, H.-Y.; Chien, Y.-C.; Lee, B.-C.; Wu, Y.-L.; Liu, Y.-P.; Wang, T.-L.; Ko, P.C.-I.; Chong, K.-M.; Wang, H.-C.; Huang, E.P.-C.; et al. Outcomes of Out-of-Hospital Cardiac Arrests after a Decade of System-Wide Initiatives Optimising Community Chain of Survival in Taipei City. *Resuscitation* **2022**, *172*, 149–158, doi:10.1016/j.resuscitation.2021.12.027.

42. MyCares^{TM} Available online: https://mycares.net/ (accessed on 23 February 2022).

43. University of Ohio Resuscitation Outcomes Consortium (ROC) Available online: https://www.ohsu.edu/school-of-medicine/emergency/resuscitation-outcomes-consortium-roc (accessed on 10 April 2022).
